# Multiproxy paleoclimate dataset from the Bednikund alpine lake in the Central Himalaya

**DOI:** 10.1016/j.dib.2021.106930

**Published:** 2021-03-03

**Authors:** Varsha Rawat, Suman Rawat, Priyeshu Srivastava, P.S. Negi, Muthusamy Prakasam, Bahadur Singh Kotlia

**Affiliations:** a Wadia Institute of Himalayan Geology, 33, G.M.S. Road, Dehradun 248 001, India; b Instituto Oceanográfico, Universidade de São Paulo, 191, Praça do Oceanográfico, São Paulo 05508-120, Brazil; c Department of Geology, Kumaun University, Nainital 263 001, India

**Keywords:** Himalaya, Lake, Paleoclimate, Grain size, Environmental magnetism, Stable carbon isotope, Indian summer monsoon, Multiproxy

## Abstract

We describe here a multiproxy dataset (grain size, environmental magnetism, stable carbon isotope, total nitrogen, and total organic carbon) generated on a ~116 cm long trench profile from the high altitude alpine Badnikund lake in the Central Himalaya. The dataset also includes environmental magnetic and organic geochemistry data on catchment soils of the Bednikund lake. The presented data is related to the research article “Middle Holocene Indian summer monsoon variability and its impact on cultural changes in the Indian subcontinent” [1]. The chronology of the Bednikund lake trench (BBK) profile is well established with seven AMS ^14^C dates. The multiproxy data is provided in tabular format in an excel file along with ages in Mendeley Data Repository. The multiproxy data can be significantly utilized for regional correlation of Indian summer monsoon (ISM) variability during the middle Holocene as well as for correlation of global climatic events. The data can also be reutilized in paleoclimate modelling for precipitation change over the past ~6000 years.

## Specifications Table

**Table:** 

Subject	Geology
Specific subject area	Holocene, Environmental magnetism, Organic geochemistry, Sedimentology, Indian summer monsoon
Type of data	Table
How data were acquired	Grain size data were analyzed using a Malvern Mastersizer 2000 Laser Particle Size Analyzer. End member modelling analysis (EMMA) was applied on grain size data using the Matlab software with the AnalySize package. Magnetic susceptibility (χ) was measured in 0.46 kHz frequency using a Bartington MS2B Laboratory Sensor. Saturation isothermal remanent magnetization (SIRM) and coercivity of remanence (B_CR_) were measured using an ASC Scientific Impulse Magnetizer (IM-10) and a Molspin Spinner Fluxgate Magnetometer. Stable organic carbon isotope (δ^13^C_org_) and Total nitrogen (TN) were measured using an Elemental Analyzer (Flash EA 2000) coupled with a Delta V plus Continuous Flow Isotope Ratio Mass Spectrometer (CF-IRMS), Thermo Scientific through ConFlow IV. Total organic carbon (TOC) was measured using a Shimadzu SSM-5000A attached with a TOC-LCPH Analyzer.
Data format	Raw and Analyzed
Parameters for data collection	Data were collected for the reconstruction of Indian summer monsoon (ISM) variability during the middle Holocene on centennial to millennial-scale. For this purpose, high-resolution sampling at ~1 cm interval up to ~116 cm depth from the peripheral margin of the Bednikund lake was carried out. Care was taken to avoid the anthropogenic disturbances. The samples were air-dried and processed in the laboratory using standard protocols for different analytical measurements.
Description of data collection	Grain size: Bulk samples (~1.5 g) were pre-treated to remove organic matter and carbonates and then measured. Environmental magnetism: Bulk samples (~10 g) were packed airtight in non-magnetic styrene pots and were measured. Organic carbon isotopes: ~1 g of homogenized carbonate free fine powdered samples were analyzed. Total organic carbon: ~10-20 mg oven-dried (~60°C) finely powdered samples were measured for total carbon (TC) and total inorganic carbon (TIC). Difference between TC and TIC was calculated as TOC. TN: ~1 g of bulk fine powdered samples were measured.
Data source location	Bedni Bugyal, Garhwal Himalaya, India (N 30.2060°, E 79.6661°) (Elevation = 3554 m asl).
Data accessibility	Mendeley Data http://dx.doi.org/10.17632/zkn8rs76hy.1
Related research article	Rawat, V., Rawat, S., Srivastava, P., Negi, P.S., Prakasam, M., Kotlia, B.S., Middle Holocene Indian summer monsoon variability and its impact on cultural changes in the Indian subcontinent. Quaternary Science Reviews 255 (2021), 106825. https://doi.org/10.1016/j.quascirev.2021.106825.

## Value of the Data

• The high resolution multiproxy data provide an opportunity to reconstruct centennial to millennial scale ISM variability. The data can also be utilized to understand the impact of precipitation variability on cultural changes in the Indian subcontinent during middle Holocene.
• The multiproxy data from the Bednikund lake give new information on the ISM variability and global climatic events during the past 6000 years. This will benefit future researchers working on Holocene paleoclimate modelling.
• These data can be used in the regional correlation of ISM variability in the monsoon dominated regions of Asia during the middle Holocene. The data can also be utilized in the paleoclimate modelling as well as in understanding the role of solar insolation in the ISM variability during the middle Holocene.

## 1 Data Description

**Fig. 1:**
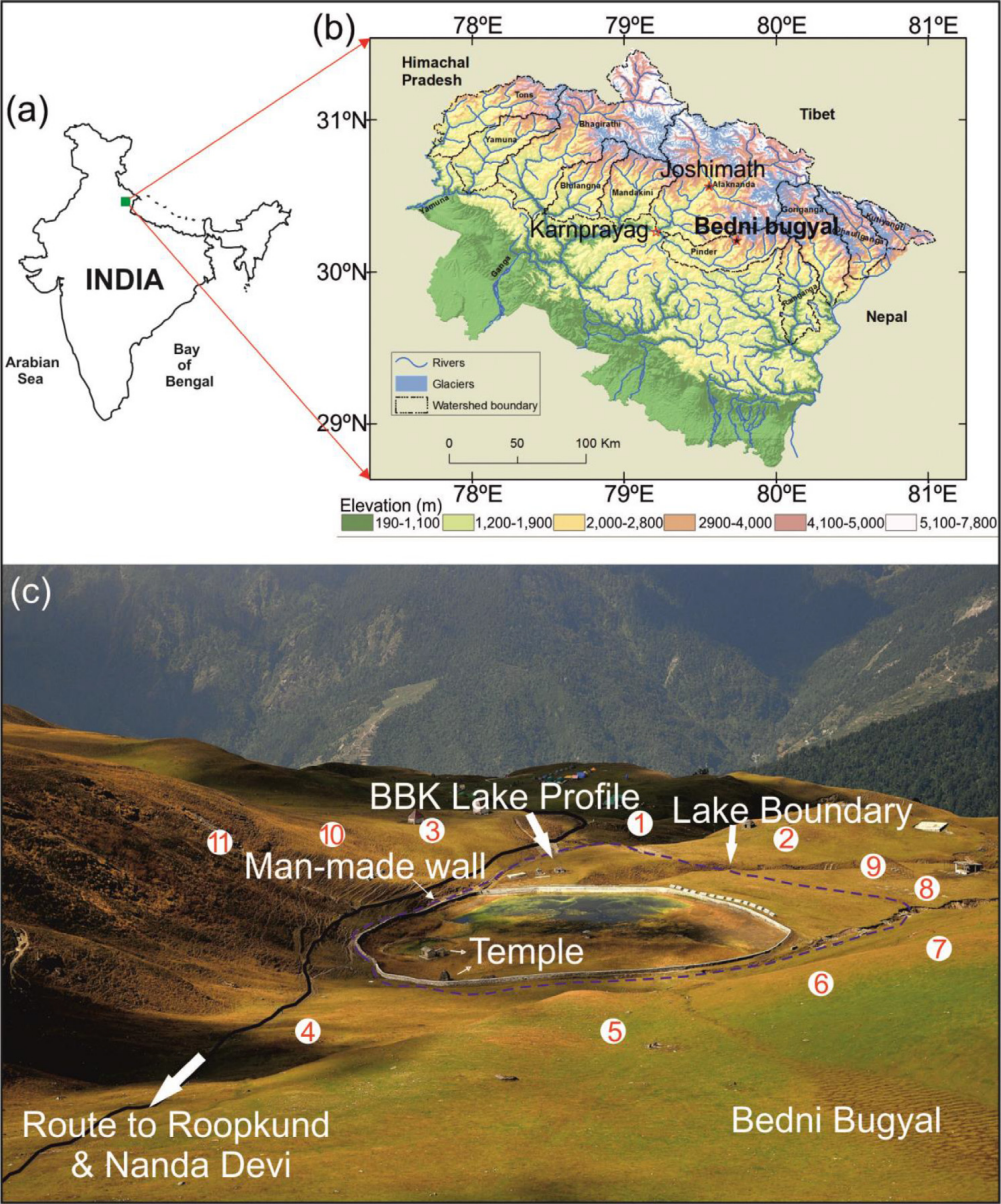
(a) The location of Garhwal basin in the map of India. (b) The watershed map of Garhwal Himalaya showing distribution of glaciers and rivers. (c) Field photograph of the Bednikund lake showing locations of lake trench profile BBK and catchment soil samples. The numbers 1 to 11 show location of the catchment soils. The Figs. 1b and 1c are adopted from [1].

This dataset describes grain size, environmental magnetism and organic geochemistry results of the Bednikund lake sediments and catchment soil samples. The data have been uploaded in the Mendeley Data repository in excel table format [2]. Fig. 1 shows sampling sites of Bednikund lake sediment (BBK) profile and catchment soils. A total of 116 samples at ~1 cm interval were collected from ~116 cm long BBK profile and a total of 11 catchment soil samples (~0-5 cm depth) were collected around the Bednikund lake (Fig. 1). The grain size data are in three fractions i.e., clay, silt and sand. The grain size data also include the end member modelling results. Three end-member results are EM1, EM2 and EM3. For different fraction composition and mean sizes of each end-members see Fig. 3 and Table 2 of Ref. [1]. The environmental magnetic results include χ, SIRM and B_CR_ data, whereas organic geochemistry results include TOC, TN and δ^13^C_org_ data. All these data have been given along with the calibrated modelled ages of the BBK profile from seven AMS^14^C dates [see Fig. 2 of Ref. 1]. The catchment soils data include environmental magnetic (χ, SIRM and B_CR_) results and organic geochemistry (TOC, TN and δ^13^C_org_) results.

## 2 Experimental Design, Materials and Methods

Lake sediments were subjected to different analytical treatments to generate high quality multiproxy data. For grain size measurement, ~1.5 g of samples were first oven-dried at ~50°C. The samples were then treated with 10 ml of 10% HCl to remove carbonate content. Following this, samples were again treated with 20 ml of 30% H_2_O_2_ to remove the organic matter. After each acid treatment, the samples were rinsed multiple times (until pH become neutral) with distilled water and centrifuged at 5000 rpm for 5 minutes to remove the acidic ions. The grain size distribution of samples was then measured with 100 bins ranging from 0.02 to 2000 μm with an obscuration level of 10±3% using a Malvern Mastersizer 2000 Laser Particle Size Analyzer. The grain size data were statistically analyzed for distinct subpopulations in the grain size components of sediments using end‐member modelling analysis in the Matlab software with the AnalySize package.

For magnetic measurements, ~10 g of air-dried bulk sediment samples were packed air-tight in the standard non-magnetic (styrene) pots. First, χ was measured in 0.46 kHz frequency using a Bartington MS2B laboratory sensor. Since samples were magnetically weak in nature, pre- and post-measurements air reading was also acquired for instrument drift corrections using formula χ (corrected) = sample χ - {(first air χ+second air χ)/2} [3,4]. After χ measurements, a forward 2200 mT field was induced in samples using an ASC Scientific Impulse magnetizer (IM-10) and remanence was measured in a Molspin spinner fluxgate magnetometer. The remanence at 2200 mT field was considered as SIRM. The samples were then induced in various backfield steps up to 500 mT for B_CR_ measurement [5].

For δ^13^C_org_ measurement, ~1 g of bulk samples were finely powdered and homogenized. The finely powdered samples were then treated with 0.6N HCl to remove the carbonate content. Subsequently, samples were rinsed repeatedly with Milli-Q water and centrifuged to remove acid and soluble salts. The samples were then oven-dried at 50°C and again fine powdered prior to δ^13^C_org_ analysis. The finely powdered samples were weighed and packed into ultra-cleaned pressed tin capsules for δ^13^C_org_ measurements. For TN content, non-acid treated bulk fine powdered samples were packed into ultra-cleaned pressed tin capsules. The δ^13^C_org_ and TN were measured using an Elemental Analyzer (Flash EA 2000) coupled with a Delta V plus Continuous Flow Isotope Ratio Mass Spectrometer (CF-IRMS), Thermo Scientific through a ConFlow IV [6]. The TN content was calculated from the peak area obtained from the sum of integrated m/z 28 and 29 signals measured on bulk samples in CF-IRMS. International (IAEA-CH3, USGS-32 KNO_3_ and ACA) and in-house standards (Sigma-Aldrich glucose, KNO_3_) were also analyzed before and after every six samples to calibrate the reference gas and carbon isotopic data, and to check the accuracy of the isotopic measurements with an external precision of + 0.1‰ (1σ). The reproducibility of data was also checked by repeated measurements of samples.

For TOC measurement, ~10-20 mg of samples were oven-dried at ~60°C and fine powdered. The samples were measured using a Shimadzu SSM-5000A attached with a TOC-LCPH analyzer. The instrument functions on the principle of oxidation through heating and combustion, and measures concentration of TC and TIC. TOC was calculated as a difference between TC and TIC and presented in weight percentages.

## Credit Author Statement

Conceptualization: SR, VR and PS; Sampling: SR and PSN; Data curation: VR and SR; Formal analysis: SR, VR, PS, PSN, PM and BSK. Writing – original draft: SR, PS and VR wrote original draft. Writing – review & editing: SR and PS. Supervision: SR.

## Declaration of Competing Interest

The authors declare that they have no known competing financial interests or personal relationships which have or could be perceived to have influenced the work reported in this article.
